# Radiotherapy Is Associated with an Accelerated Risk of Ischemic Stroke in Oral Cavity Cancer Survivors after Primary Surgery

**DOI:** 10.3390/cancers12030616

**Published:** 2020-03-06

**Authors:** Feng-Che Kuan, Kuan-Der Lee, Shiang-Fu Huang, Ping-Tsung Chen, Cih-En Huang, Ting-Yao Wang, Min-Chi Chen

**Affiliations:** 1 Department of Hematology and Oncology, Chang Gung Memorial Hospital, Chiayi, Chiayi 613, Taiwan; 8902029@cgmh.org.tw (F.-C.K.); ptchen@cgmh.org.tw (P.-T.C.); 8802058@cgmh.org.tw (C.-E.H.); yukishirochy@cgmh.org.tw (T.-Y.W.); 2 Division of Hematology and Oncology, Department of Internal Medicine, Taipei Medical University Hospital, and School of Medicine, College of Medicine, Taipei Medical University, Taipei 110, Taiwan; kdlee@h.tmu.edu.tw; 3 Department of Otolaryngology & Head and Neck Surgery, Chang Gung Memorial Hospital, Linkou, Taoyuan 333, Taiwan; bigmac@cgmh.org.tw; 4 Department of Public Health, Biostatistics Consulting Center, College of Medicine, Chang Gung University, Guishan, Taoyuan 333, Taiwan

**Keywords:** oral cavity carcinoma, ischemic stroke, survivor

## Abstract

The number of oral cavity carcinoma (OCC) survivors continues to increase due to advances in definitive surgery and radiation therapy (RT), however the risk of ischemic stroke is unclear in long-term survivors. In this study, survivors are defined as those who survived for >5 years after a diagnosis of OCC. They were matched at a 1:5 ratio with normal controls. Those who received surgery alone versus surgery+RT were also matched at a 1:1 ratio. From 2000 to 2005, 5172 OCC survivors who received surgery alone (*n* = 3205) or surgery+RT (*n* = 1967), and 25,860 matched normal controls were analyzed using stratified Cox regression models. Adjusted HRs (aHR) revealed that the surgery+RT group (aHR = 1.68, *p* < 0.001) had an elevated risk of stroke, but this was not seen in the surgery alone group (aHR = 0.99, *p* = 0.953). Furthermore, the age at stroke onset was at least 10 years earlier in the surgery+RT group than in the controls. In conclusion, radiotherapy increased the risk of ischemic stroke by 68% and also accelerated the onset of stroke in long-term OCC survivors after primary surgery compared with matched normal controls. Secondary prevention should include stroke as a late complication in OCC survivorship programs.

## 1. Introduction

Oral cavity carcinoma (OCC) comprises 38.2% of all head and neck cancers and remains a main cause of morbidity and mortality in Taiwan, and accounts for 1.5% of all cancer cases globally [1]. Although it shares the same risk factors as oropharyngeal and laryngeal carcinomas such as smoking tobacco, alcohol consumption, and betel nut chewing, OCC remains primarily a surgical disease and adjuvant radiation is given to high-risk patients with or without chemotherapy, all of which contribute to an increased survival rate [2]. Due to advances in surgical and radiation technology, the number of male OCC survivors is expected to increase from around 250,000 people in 2020 to over 300,000 in 2030 [3]. Previous studies have suggested that there may be an increased risk of stroke in patients with head and neck cancers, and have proposed that radiation may cause this long-term complication [4,5,6,7,8,9]. However, head and neck cancer is a heterogeneous disease, and these studies have included all patients from diagnosis rather than specifically focusing on long-term survivors. Thus, we conducted this population-based cohort study to investigate the association between radiation therapy (RT) and ischemic stroke, specifically in long-term OCC survivors, defined as patients who had survived for longer than 5 years after diagnosis.

## 2. Results

### 2.1. Characteristics of Study Participant

From 2000 to 2005, a total of 5172 eligible 5-year OCC survivors were identified from the Registry of Catastrophic Illnesses (RCI), and 25,860 normal controls matched by sex, age at index date, urbanization, and income-related insurance payment were selected from the Longitudinal Health Insurance Database 2005 (LHID2005). The median follow-up time after the index date was 4.07 years. The baseline characteristics are listed in Table 1. Among the OCC survivors, 89.8% were male and the mean age at the index date was 55.28 (± 11.02) years. Most of the OCC survivors lived in the least urbanized areas (34.6%) and had the lowest income-related insurance payments (69.3%). The matched normal cohort was comparable to the OCC survivors with respect to sex, age, urbanization, and income-related insurance payment (all *p* ≥ 0.718). However, the OCC survivors had lower rates of most comorbidities including hypertension (21.4 vs. 25.5%, *p* < 0.001), ischemic heart disease (4.9 vs. 8.6%, *p* < 0.001), atrial fibrillation (0.5 vs. 0.8%, *p* = 0.021), peripheral arterial occlusive disease (0.1 vs. 0.5%, *p* = 0.001), hyperlipidemia (4.4 vs. 7.3%, *p* < 0.001), and chronic kidney disease (0.2 vs. 0.6%, *p* = 0.011) than the matched controls. 

### 2.2. Risk Factors Related to Ischemic Stroke: Comparison between the OCC Survivors and Matched Normal Control

**Figure 1:**
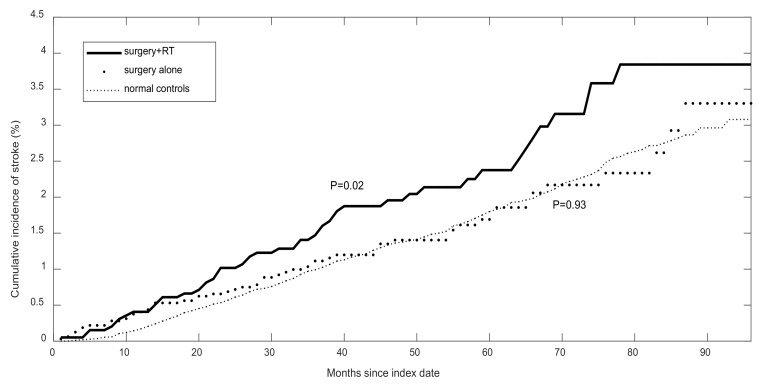
Cumulative incidence rate of ischemic stroke in the oral cavity cancer survivors with two treatment modalities and normal controls.

The overall stroke incidence in the OCC survivors was similar to that in the matched controls (457.85 vs. 375.43 per 100,000 person-years, *p* = 0.074, Table 1). However, when the treatment-specific incidence rate was compared to the normal controls, the OCC survivors who received surgery and RT had a much higher risk of stroke (588.03 vs. 375.43 per 100,000 person-years, *p* = 0.003). In contrast, the OCC survivors who received surgery alone and the normal controls had similar incidence rates (382.72 vs. 375.43 per 100,000 person-years, *p* = 0.895). These results were consistent with the cumulative incidence curves (Figure 1). The OCC survivors who received surgery with RT had a higher cumulative incidence than the normal controls (*p* = 0.02), but there was no significant difference between the OCC survivors who received surgery alone and the controls (*p* = 0.93). Furthermore, the univariate analysis showed that only the OCC survivors who received surgery plus RT had a significant risk of stroke (HR = 1.53, *p* = 0.004), and those who had any one of the following comorbidities had a higher risk of stroke: diabetes (HR = 1.82, *p* < 0.001), hypertension (HR = 2.15, *p* < 0.001), ischemic disease (HR = 1.37, *p* = 0.013), atrial fibrillation (HR = 2.36, *p* = 0.001), and chronic kidney disease (HR = 3.33, *p* = 0.007) (left panel, Table 2). However, when treatment modalities and significant comorbidities were considered simultaneously, the aHRs revealed that only the surgery plus RT group had an elevated risk (aHR = 1.68, *p* < 0.001), but not the surgery alone group (aHR = 0.99, *p* = 0.953) compared to the normal controls (right panel, Table 2). In addition, to determine the impact of chemotherapy on stroke, a distinction was made between patients who received adjuvant radiotherapy and those who received adjuvant chemoradiotherapy. We found the risk of stroke between surgery with RT alone (n = 883) and surgery with chemoradiotherapy (n = 1084) did not show any significant differences (aHR = 1.64 vs. 1.72, *p* = 0.879). Therefore, they were combined as surgery with the RT group (n = 1967) for further analysis (Table 2).

### 2.3. Risk Factors Related to Ischemic Stroke: Comparison between Treatment Modalities in the OCC Survivors

Each pair of OCC survivors included one patient from the surgery with the RT group and one patient from the surgery alone group. Except for matching variables (sex, age, urbanization and income-related insurance payment), they were also comparable with respect to comorbidities of peripheral arterial occlusive disease and chronic kidney disease (Table 3). However, the surgery with RT group had a higher incidence of atrial fibrillation (0.61% vs. 0.15%, *p* = 0.020) but lower incidence rates of diabetes (10.70% vs. 14.13%, *p* = 0.001), hypertension (16.33% vs. 23.35%, *p* < 0.001), ischemic heart disease (3.64% vs. 5.07%, *p* = 0.027), and hyperlipidemia (2.82% vs. 4.61%, *p* = 0.003) than the surgery alone group. The crude incidence rates of ischemic stroke and death per 100,000 person-years revealed that the survivors in the surgery with RT group were more likely to develop ischemic stroke or die than the surgery alone group (stroke: 591.66 vs. 362.73, *p* = 0.034; death: 4397.55 vs. 1207.91, *p* < 0.001). The elevated risks of stroke and death in the surgery with the RT group persisted after adjusting for comorbidities (Table 4). Specifically, the OCC survivors who received surgery and RT were at a higher risk of stroke and death than those who received surgery alone (stroke: aHR = 1.48, *p* = 0.048; death: aHR = 4.341, *p* < 0.001). 

### 2.4. Premature Stroke in the OCC Survivors

**Figure 2:**
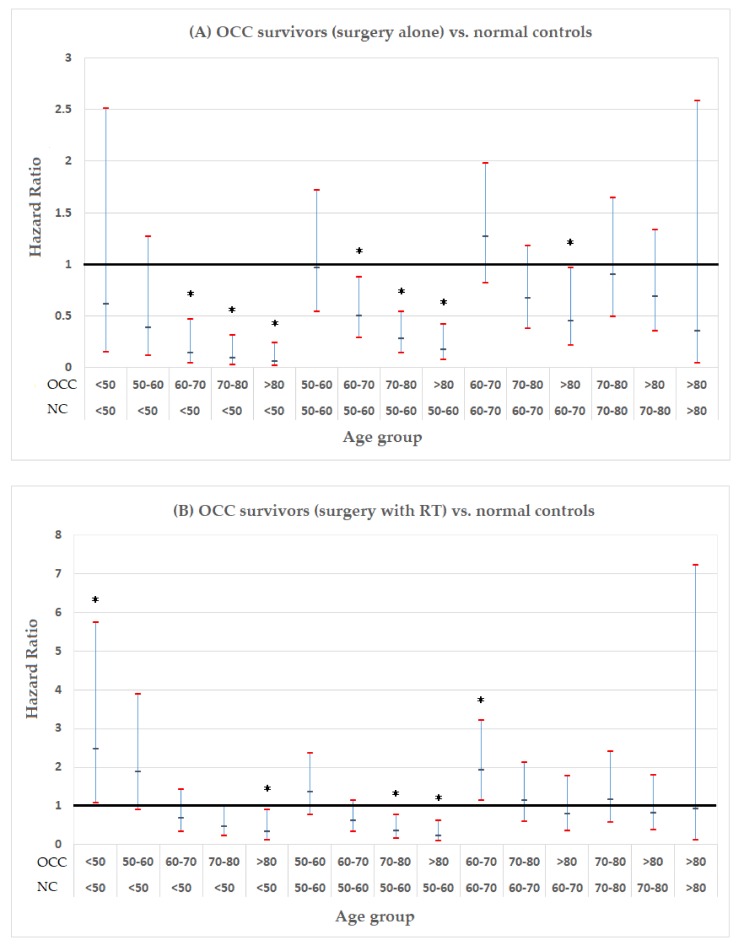
Adjusted hazard ratios with 95% confidence intervals: survivors of oral cavity cancer (OCC) versus normal controls (NC) in different age groups (* *p* < 0.05, reference group: normal controls). (**A**) OCC survivors with surgery alone vs. normal controls; (**B**) OCC survivors with surgery and RT vs. normal controls.

After controlling for basic characteristics (sex, urbanization level, income-related insurance payment) and comorbidities, the OCC survivors who received surgery alone had a comparable age at stroke onset to the normal controls at similar age strata (Figure 2A). However, the age at stroke onset was at least 10 years earlier in the surgery with the RT group than in the controls, especially for the young patients (Figure 2B). For example, in the surgery with RT group, survivors <50 years old had a higher adjusted risk of stroke than the controls aged <50 years, but a similar risk to the controls aged 50 to 80 years. In addition, the risk in the survivors aged 50 to 60 years was equivalent to that in the controls aged up to 70 years. These results showed that premature stroke was a complication in the OCC survivors who received surgery and RT in all age groups.

## 3. Discussion

In this study, we found that radiation increased the risk of ischemic stroke by 68%, and also accelerated the occurrence of stroke by more than 10 years in OCC survivors compared to matched normal controls. Huang et al. reported a single-center study in which radiation increased the risk of stroke compared to surgery alone in younger patients (age <55 years at diagnosis) with head and neck cancers [5]. In the current population-based study we focused on OCC survivors (>5 years), and found that the incidence rate of stroke in the patients overall was similar to that in the matched-controls (HR = 1.18, *p* = 0.111, Table 2). However, the risk was significantly higher in the survivors who received surgery with radiation (aHR = 1.68, *p* < 0.001) compared to the normal controls, but not in the surgery alone group (aHR = 0.99, *p* = 0.953). In our cohort, the cumulative incidence rates of stroke were similar between the survivors who received surgery alone and the normal controls (*p* = 0.93, Figure 1) and higher in the survivors who received surgery with radiation (*p* = 0.02), indicating that radiation could be a risk factor for stroke. To further adjust for the effect of other confounding factors including smoking, alcohol use and betel quid chewing [6], we matched the surgery and the RT group with the surgery alone group by gender, age, urbanization and income-related insurance payment. The results showed that the surgery with the RT group had increased risks of ischemic stroke and death (*p* = 0.034 and *p* < 0.001, respectively, Table 3). Therefore, radiation was an independent risk factor for stroke. 

The radiation field for OCC is in the proximity of the carotid arteries, and the effect of ionizing radiation on plaque formation has been reported [10]. Despite the introduction of intensity-modulated RT in Taiwan*,* the late effects of radiation resulting in vascular injury are inevitable due to radiation scattering. Radiation-induced atherosclerosis of the carotid artery is a clinically relevant late complication. In this study, radiation, but not surgery, posed a higher risk of ischemic stroke in the 5-year OCC survivors. Stenosis of the common and internal carotid arteries has been associated with the time interval from RT [11], and this late effect related to radiation may have been missed in the OCC survivors since their cancer had been cured for 5 years or more. However, late complications such as ischemic stroke will have a significant impact on survivorship and pose a public health burden. In addition to radiation, hypertension, atrial fibrillation and diabetes were important risk factors for ischemic stroke or death in the OCC patients in this study. Radiation may accelerate the pathologic aging process on blood vessels and also promote the risk of stroke. This accelerated effect may be validated by our findings, in that the age at stroke onset was at least 10 years earlier in the OCC survivors who received surgery and RT compared to the normal population, especially for the young patients (Figure 2B). These results provide evidence that young OCC survivors (<50 years old) should not be exempt from stroke surveillance. Similar findings have been reported in nasopharyngeal carcinoma survivors, in whom radiation is the main treatment modality [7,8,9].

There are several limitations to this study. First, some potential confounding factors were not available for analysis, including data on human papillomavirus and radiotherapy-induced toxicity which have been shown to be associated with cerebrovascular events following RT [12,13,14]. Smoking is also an important risk factor for stroke [15]. However, the impact of this modifiable factor could not be evaluated in our study. To eliminate the confounding effect of smoking, we adjusted for smoking-associated comorbidities including hypertension, ischemic heart disease, atrial fibrillation, peripheral arterial occlusive disease, hyperlipidemia and chronic kidney disease in the statistical models while comparing OCC survivors with normal controls. We also conducted a matched study in OCC survivors as smoking is very common among them. Second, one-third of the patients in this study received surgery plus RT, and among them, over half also received concomitant chemotherapy. However, the details of treatment such as volume and duration of radiation, and dose intensity of chemotherapy were not provided in the NHIRD. This prevented us from observing if there is a dose-response relationship between single-, dual-, and tri-modality therapy with regard to stroke and death in OCC survivors. Third, TNM staging is not available in both RCI and NHIRD databases, despite the fact that differences in T- or N-stages could result in different treatment modalities, such as radiation followed by surgery versus surgery alone. Further prospective studies are needed to elucidate this issue. 

## 4. Materials and Methods

### 4.1. Data Sources

The data used in this study were derived from the RCI and LHID2005, which are two subsets of records from the Taiwan National Health Insurance Research Database (NHIRD). The NHIRD is a nationwide database containing longitudinal medical records of beneficiaries enrolled in the National Health Insurance (NHI) program, which provides comprehensive health care coverage for over 98% of the Taiwanese population. The RCI database was used to identify patients with OCC or other cancers. Patients with a catastrophic illness certificate are exempt from copayments for medical services, and they are evaluated by a panel of specialists through a strict process of review of medical records, imaging, and pathology reports. The LHID2005 contains original claims data for 1,000,000 beneficiaries randomly sampled from the entire population in 2005. All data on comorbidities and treatment modalities (for the OCC cases) from 1995 to 2012 were available for analysis from inpatient and outpatient databases. This study was approved by the Institutional Review Board of Chang Gung Medical Foundation (approval number, 201600205B0).

### 4.2. Study Design

The primary endpoint in this study was ischemic stroke during the follow-up period (2005–2012). Ischemic stroke was identified if the participant was hospitalized with a major or minor diagnosis of the following International Classification of Diseases, Ninth Revision, Clinical Modification (ICD-9-CM) codes: 433.xx (occlusion of cerebral arteries), 434.xx (stenosis of precerebral arteries), 436.xx (acute but ill-defined cerebrovascular disease), or 437.1 (other generalized ischemic cerebrovascular disease), accompanied by either computed tomography or magnetic resonance imaging examinations. 

OCC survivors (ICD-9-CM codes: 140–143, 144–145) were defined as those who survived for longer than 5 years after the diagnosis of OCC, and who received surgery alone or with RT within 3 months of primary surgery, regardless chemotherapy. The first day after 5 years of survivorship was defined as the index date. To examine the risk of RT related to ischemic stroke, two matched studies were conducted: (i) all OCC survivors were matched to normal controls at a ratio of 1:5, and (ii) the OCC survivors who received surgery alone were matched at a ratio of 1:1 to OCC survivors who received surgery with RT. 

The OCC group initially were comprised of 9258 patients who were diagnosed between 2000 and 2005. However, 4086 patients were excluded due to any one of the following criteria: age <20 or >80 years at the diagnosis of OCC, survived for less than 5 years, developed second cancers or metastasis during follow-up, ischemic stroke occurred before the index date, with incomplete individual information, or received treatment modalities other than surgery alone or surgery combined with RT. Finally, 5172 OCC survivors who received surgery alone (*n* = 3205) or surgery with RT (*n* = 1967) were enrolled for analysis. Normal controls were matched using propensity scores, calculated as the probability of being a case (OCC) according to baseline variables, including age at index date (<35, 35–40, ..., 65–70, ≥70 years), sex, urbanization level (5 levels), and income-related insurance payment (5 levels). With a match ratio of 1:5, five corresponding normal controls were selected for each OCC case based on the closest propensity score. Thus, a total of 25,860 controls aged 25–85 years without any history of cancers or ischemic stroke before the index date were selected from the LHID2005. The index date of each control was assigned to be the same as that of the corresponding OCC survivor. The time to stroke in both groups was defined as the number of years from the index date to a new diagnosis of ischemic stroke, to withdrawal from the NHI program (mostly owing to death, and a few owing to imprisonment, immigration and others), or to 31 December 2012, whichever occurred first. 

The second matching procedure was performed among OCC survivors. For each OCC survivor who received surgery with RT, one corresponding OCC survivor who received surgery alone was selected based on propensity score which was calculated using the same method as for the matched OCC-control study above. Finally, 1953 paired samples were analyzed. Comorbidities associated with ischemic stroke including diabetes, hypertension, hyperlipidemia, ischemic heart disease, atrial fibrillation, peripheral arterial occlusive disease, and chronic kidney disease, and their diagnoses were defined as at least three clinical visits or at least one hospitalization during the 12 months prior to the index date. 

### 4.3. Statistical Analysis

Demographic data and comorbidities of the OCC survivors and matched controls are presented as frequency with percentage or mean with standard deviation. Because both study designs were matched, comparisons between the two groups in baseline characteristics were assessed using generalized estimating equations (GEE) [16], which took into account correlations within each cluster (one OCC case and five matched normal controls, or one OCC case with surgery alone and one OCC case with surgery and RT). A stratified Cox proportional hazards model was used to evaluate the risk of ischemic stroke and death between groups, and the results are presented as crude hazard ratios (HRs) and adjusted HRs (aHRs) with *p*-values. Specifically, when death was analyzed as an endpoint in the OCC survivors, the occurrence of ischemic stroke was treated as a time-dependent variable. In addition, the cumulative incidence rates of ischemic stroke were calculated and compared between the OCC survivors and normal controls by applying a competing risk model proposed by Kalbfleisch and Prentice [17] and Gray [18]. Data were managed and analyzed using SAS version 9.4 (SAS Institute, Inc., Cary, NC, USA). All statistical tests were two-sided, and *p* < 0.05 was considered to indicate statistical significance.

## 5. Conclusions

Radiation promoted the aging process in blood vessels and accelerated the risk of ischemic stroke in the OCC survivors after primary surgery. Therefore, secondary prevention should include stroke as a late complication for surveillance in cancer survivorship programs which may incorporate regular follow-up by carotid artery sonography and early interventions such as anti-coagulants or stent insertion [19,20].

## Figures and Tables

**Table 1:** Characteristics of the oral cavity cancer survivors and matched controls.

Characteristic	Oral Cavity Cancer (*n* = 5172)	Controls (*n* = 25,860)	*p*-Value
Male	4647 (89.8%)	23,235 (89.8%)	1.000
Age at index date (years)	55.28 ± 11.02	55.33 ± 11.36	0.718
25–35	87 (1.7%)	435 (1.7%)	
35–45	779 (15.1%)	3895 (15.1%)	
45–55	1736 (33.6%)	8688 (33.6%)	
55–65	1477 (28.6%)	7377 (28.6%)	
>65	1093 (21.1%)	5465 (21.1%)	
Urbanization			0.746
1 (least urbanized)	1788 (34.6%)	8948 (34.6%)	
2	1214 (23.5%)	6054 (23.4%)	
3	1490 (28.8%)	7466 (28.9%)	
4 (most urbanized)	680 (13.1%)	3392 (13.1%)	
Payroll-related insurance payment			1.000
1 (lowest)	3584 (69.3%)	17,920 (69.3%)	
2	692 (13.4%)	3460 (13.4%)	
3	643 (12.4%)	3,15 (12.4%)	
4 (highest)	253 (4.9%)	1265 (4.9%)	
Comorbidity			
Diabetes	692 (13.4%)	3,059 (11.8%)	0.001
Hypertension	1109 (21.4%)	6603 (25.5%)	<0.001
Ischemic heart disease	255 (4.9%)	2220 (8.6%)	<0.001
Atrial fibrillation	25 (0.5%)	203 (0.8%)	0.021
Peripheral arterial occlusive disease	7 (0.1%)	125 (0.5%)	0.001
Hyperlipidemia	228 (4.4%)	1896 (7.3%)	<0.001
Chronic kidney disease	12 (0.2%)	148 (0.6%)	0.011
Ischemic stroke (ICD-9: 433.x, 434.x, 436.x and 437.1)	100 (1.90%) 457.85 ^b^	429 (1.7%) 375.43 ^b^	0.074
Surgery alone (n = 3205)	53 ^c^ (1.65%) 382.72 ^b^		0.895 ^d^
Surgery + radiation therapy ^a^ (n = 1967)	47 ^c^ (2.39%) 588.03 ^b^		0.003 ^d^

Data are presented as mean ± SD or *n* (%). Generalized estimating equations were used to compare variables between the survivors and normal controls who were matched by 5-year age group, sex, urbanization level, and income-related insurance payment. ^a^ Radiation therapy was done within 3 months before or after surgery. ^b^ Incidence rate per 100,000 person-years. ^c^ Number of ischemic strokes in the surgery alone group and surgery+RT group, respectively. ^d^ Significance of crude incidence compared with the controls.

**Table 2:** Crude and adjusted hazard ratios for the occurrence of ischemic stroke.

Variables	Crude HR (95% CI)		*p*-Value	Adjusted HR ^a^ (95% CI)		*p*-Value
Group: normal controls	1			1		
OCC survivors						
Surgery alone	0.97	(0.74–1.28)	0.848	0.99	(0.75–1.31)	0.953
Surgery + RT ^b^	1.53	(1.14–2.06)	0.004	1.68	(1.26–2.25)	<0.001
w/ chemotherapy ^c^				1.64	(1.07–2.25)	0.021
w/o chemotherapy ^c^				1.72	(1.15–2.55)	0.007
Comorbidity						
Diabetes	1.82	(1.48–2.23)	<0.001	1.46	(1.18–1.81)	<0.001
Hypertension	2.15	(1.78–2.59)	<0.001	2.00	(1.65–2.43)	<0.001
Ischemic heart disease	1.37	(1.06–1.76)	0.013			
Atrial fibrillation	2.36	(1.40–3.96)	0.001	2.02	(1.17–3.46)	0.009
Peripheral arterial occlusive disease	1.84	(0.78–4.34)	0.159			
Hyperlipidemia	1.09	(0.80–1.48)	0.595			
Chronic kidney disease	3.33	(1.37–8.11)	0.007			

OCC: oral cavity cancer; RT: radiation therapy; HR: hazard ratio. ^a^ Adjusted HRs and *p*-values were obtained from a stratified Cox model which included significant explanatory variables and withdrawal was treated as a competing risk. ^b^ Among 1967 survivors in the group of surgery and RT, 1084 also received chemotherapy and 883 did not. ^c^ Treatment modalities examined in the Cox model were surgery alone, surgery with RT, and surgery with chemoradiotherapy. The difference in risk between with and without chemotherapy in the RT group was not significant (aHR: 1.64 vs. 1.72, *p* = 0.879).

**Table 3:** Characteristics of the oral cavity survivors in 1:1 matching of those who received surgery with radiation therapy and those who received surgery alone.

Variables	Treatment Modality		*p*-Value
	Surgery + Radiation Therapy (n = 1953)	Surgery alone (n = 1953)	
Male	1795 (91.91%)	1795 (91.91%)	1.000
Age at index date (years)			1.000
25–45	363 (18.59%)	363 (18.59%)	
45–55	697 (35.69%)	697 (35.69%)	
55–65	551 (27.70%)	551 (27.70%)	
>65	352 (18.02%)	352 (18.02%)	
Urbanization			0.677
1 (least urbanized)	711 (36.41%)	714 (36.56%)	
2	460 (23.55%)	457 (23.40%)	
3	554 (28.37%)	555 (28.42%)	
4 (most urbanized)	228 (11.67%)	227 (11.62%)	
Payroll-related insurance payment			0.532
1 (lowest)	1409 (72.15%)	1407 (72.04%)	
2	248 (12.70%)	252 (12.90%)	
3	217 (11.11%)	217 (11.11%)	
4 (highest)	79 (4.05%)	77 (3.94%)	
Comorbidity			
Diabetes	209 (10.70%)	276 (14.13%)	0.001
Hypertension	319 (16.33%)	456 (23.35%)	<0.001
Ischemic heart disease	71 (3.64%)	99 (5.07%)	0.027
Atrial fibrillation	12 (0.61%))	3 (0.15%)	0.020
Peripheral arterial occlusive disease	1 (0.05%)	4 (0.20%)	0.180
Hyperlipidemia	55 (2.82%)	90 (4.61%)	0.003
Chronic kidney disease	4 (0.20%)	2 (0.10%)	0.414
Ischemic Stroke	47 (2.41%) 591.66 ^a^	31 (1.59%) 362.73 ^a^	0.034
Death	354 (18.13%) 4397.55 ^a^	104 (5.33%) 1207.91 ^a^	<0.001

Gender, 5-year age group, urbanization and income-related insurance payment were matched in a propensity score model. ^a^ incidence rate per 100,000 person-years.

**Table 4:** Adjusted hazard ratios for ischemic stroke and death as endpoints in matched oral cavity survivors (*n* = 3906).

Endpoint	Adjusted HR	95% CI	*p*-Value
**Ischemic stroke**			
Treatment modality			
Surgery+RT	1.48	(1.01–2.20)	0.048
Surgery alone	1		
Comorbidity			
Hypertension	1.91	(1.04–3.51)	0.034
**Death**			
Treatment modality			
Surgery+RT	4.34	(3.43–5.49)	<0.001
Surgery alone	1		
Comorbidity			
Diabetes	1.81	(1.19–2.75)	0.004

RT: radiation therapy; HR: hazard ratio; CI: confidence interval.

## References

[1] Shield K.D., Ferlay J., Jemal A., Sankaranarayanan R., Chaturvedi A.K., Bray F., Soerjomataram I. (2017). The global incidence of lip, oral cavity, and pharyngeal cancers by subsite in 2012. CA Cancer J. Clin..

[2] Chinn S.B., Myers J.N. (2015). Oral cavity carcinoma: Current management, controversies, and future directions. J. Clin. Oncol..

[3] Miller K.D., Nogueira L., Mariotto A.B., Rowland J.H., Yabroff K.R., Alfano C.M., Jemal A., Kramer J.L., Siegel R.L. (2019). Cancer treatment and survivorship statistics, 2019. CA Cancer J. Clin..

[4] Arthurs E., Hanna T.P., Zaza K., Peng Y., Hall S.F. (2016). Stroke after radiation therapy for head and neck cancer: What is the risk?. Int. J. Radiat. Oncol. Biol. Phys..

[5] Huang Y.S., Lee C.C., Chang T.S., Ho H.C., Su Y.C., Hung S.K., Lee M.S., Chou P., Chang Y.H., Lee C.C. (2011). Increased risk of stroke in young head and neck cancer patients treated with radiotherapy or chemotherapy. Oral Oncol..

[6] Ko Y.C., Huang Y.L., Lee C.H., Chen M.J., Lin L.M., Tsai C.C. (1995). Betel quid chewing, cigarette smoking and alcohol consumption related to oral cancer in Taiwan. J. Oral Pathol. Med..

[7] Chu C.N., Chen P.C., Bai L.Y., Muo C.H., Sung F.C., Chen S.W. (2013). Young nasopharyngeal cancer patients with radiotherapy and chemotherapy are most prone to ischemic risk of stroke: A national database, controlled cohort study. Clin. Otolaryngol..

[8] Lee C.C., Su Y.C., Ho H.C., Hung S.K., Lee M.S., Chiou W.Y., Chou P., Huang Y.S. (2011). Increase risk of ischemic stroke in young nasopharyngeal carcinoma patients. Int. J. Radiat. Oncol. Biol. Phys..

[9] Chen M.C., Kuan F.C., Huang S.F., Lu C.H., Chen P.T., Huang C.E., Want T.Y., Chen C.C., Lee K.D. (2019). Accelerated risk of premature ischemic stroke in 5-year-survivors of nasopharyngeal carcinoma. Oncologist.

[10] Kloosterman A., Dillen T.V., Bijwaard H., Heeneman S., Hoving S., Stewart F.A., Dekkers F. (2017). How radiation influences atherosclerotic plaque development: A biophysical approach in ApoE⁻/⁻ mice. Radiat. Environ. Biophys..

[11] Cheng S.W., Wu L.L., Ting A.C., Lau H., Lam L.K., Wei W.I. (1999). Irradiation-induced extracranial carotid stenosis in patients with head and neck malignancies. Am. J. Surg..

[12] Addision D., Seidelmann S.B., Janjua S.A., Emami H., Staziaki P.V., Hallett T.R., Szilveszter B., Lu M.T., Cambria R.P., Hoffmann U. (2019). Human papillomavirus status and the risk of cerebrovascular events following radiation therapy for head and neck cancer. J. Am. Heart. Assoc..

[13] Hwang T.Z., Hsiao J.R., Tsai C.R., Chang J.S. (2015). Incidence trends of human papillomavirus-related head and neck cancer in Taiwan, 1995–2009. Int. J. Cancer.

[14] Blanchard P., Belkhir F., Temam S., El Khoury C., De Felice F., Casiraghi O., Patrikidou A., Mirghani H., Levy A., Even C. (2017). Outcomes and prognostic factors for squamous cell carcinoma of the oral tongue in young adults: A sincle-institution case-matched analysis. Eur. Arch. Otorhinolarygnol..

[15] Sacco R.L. (1995). Risk factors and outcomes for ischemic stroke. Neurology.

[16] Liang K.Y., Zeger S.L. (1986). Longitudinal data analysis using generalized linear models. Biometrika.

[17] Kalbfleisch J.D., Prentice R. (2002). The Analysis of Failure Time Data.

[18] Gray R.A. (1988). Class of K-sample tests for comparing the cumulative incidence of a competing risk. Ann. Stat..

[19] Mohammadkarim A., Mokhtari-Dizaji M., Kazemian A., Saberi H. (2018). Hemodynamic analysis of radiation-induced damage in common carotid arteries by using color Doppler ultrasonography. Ultrasonography.

[20] Sharma M., Hart R.G., Connolly S.J., Bosch J., Shestakovska O., Ng K.K.H., Catanese L., Keltai K., Aboyans V., Alings M. (2019). Stroke Outcomes in the COMPASS Trial. Circulation.

